# MicroRNA: Important Player in the Pathobiology of Multiple Myeloma

**DOI:** 10.1155/2014/521586

**Published:** 2014-06-03

**Authors:** Chonglei Bi, Wee Joo Chng

**Affiliations:** ^1^Experimental Therapeutics, Cancer Science Institute of Singapore, No. 12-02, Centre for Translational Medicine, 14 Medical Drive, Singapore 117599; ^2^Yong Loo Lin School of Medicine, NUHS Tower Block level 11, 1E Kent Ridge Road, Singapore 119228; ^3^Department of Hematology-Oncology, National University Cancer Institute, National University Health System, 1E, Kent Ridge Road, Singapore 119228

## Abstract

Recent studies have revealed a pivotal role played by a class of small, noncoding RNAs, microRNA (miRNA), in multiple myeloma (MM), a plasma cell (PC) malignancy causing significant morbidity and mortality. Deregulated miRNA expression in patient's PCs and plasma has been associated with tumor progression, molecular subtypes, clinical staging, prognosis, and drug response in MM. A number of important oncogenic and tumor suppressor miRNAs have been discovered to regulate important genes and pathways such as p53 and IL6-JAK-STAT signaling. miRNAs may also form complex regulatory circuitry with genetic and epigenetic machineries, the deregulation of which could lead to malignant transformation and progression. The translational potential of miRNAs in the clinic is being increasingly recognized that they could represent novel biomarkers and therapeutic targets. This review comprehensively summarizes current progress in delineating the roles of miRNAs in MM pathobiology and management.

## 1. Multiple Myeloma

Multiple myeloma (MM) is a tumor of antibody-secreting plasma cells (PCs) characterized by the clonal expansion and accumulation of monotypic PCs in the bone marrow (BM) [1]. It causes about 1% of neoplastic diseases and 13% of hematological malignancies [2]. Clinically, MM patients exhibit one or more symptoms including lytic bone disease, hypercalcemia, anemia, and compromised renal functions [3]. MM is always preceded by an asymptomatic premalignant stage called monoclonal gammopathy of undetermined significance (MGUS), which progresses to myeloma or related malignancies at a rate of 1% per year [4, 5]. Patients with MGUS are by definition symptom-free, but with measurable concentration of monoclonal protein or have an abnormality in serum-free light chain assay [6]. Although MM cells are strongly dependent on BM microenvironment, more aggressive tumors may extend to extramedullary sites. Extramedullary MM (EMM) can also present with a leukemic phase which can be classified as primary plasma cell leukemia (pPCL) if it arises* de novo*, or secondary PCL if preceded by intramedullary MM [7, 8]. Most of the human MM cell lines (HMCLs) are generated from EMM or PCL tumors [9]. The Durie-Salmon staging system which mainly reflects tumor burden was the first commonly used staging system for MM [10]. It has been superseded by the International Staging System (ISS), a 3-group classification based on two simple and routine laboratory tests widely available [11, 12].

MM is characterized by complex genetic and epigenetic abnormalities [13, 14]. Biologically, myeloma can be broadly divided into hyperdiploid and nonhyperdiploid categories, each consists of about half of MGUS and MM tumors. Hyperdiploid myeloma (H-MM) is characterized by multiple trisomies involving odd number chromosomes except chromosome 13 and a lower prevalence of primary translocation involving the immunoglobulin heavy chain (IgH) locus at 14q32, whereas nonhyperdiploid myeloma (NH-MM) is characterized by IgH translocations, most commonly t(4; 14) which translocates MMSET and FGFR3 at 4p16.3 to the IgH enhancers, t(11; 14) involving CCND1, and t(14; 16) involving MAF [15, 16]. H-MM and NH-MM are observed in both MGUS and MM, suggesting that they are early oncogenic events although high-risk MM is more common in NH-MM cases [17]. Another early and perhaps unifying event in MGUS and MM is the upregulation of cyclin D genes independent of the H-MM and NH-MM dichotomy, although it does not appear to be associated with increased proliferation [8]. In addition, a number of secondary genetic lesions associated with disease progression and survival have been identified, including activating mutations in RAS and BRAF, increasing frequency of MYC overexpression in disease progression, chromosomal 13 deletion, chromosome 17p loss, and p53 abnormalities, chromosome 1 abnormalities, IL6-JAK-STAT3 and NF*κ*B activation. Notably, the frequency of 17p loss increases as disease progresses and has been uniformly accepted as a marker for high-risk MM [18–27].

Studies have investigated the molecular basis for MM progression using gene expression profiling (GEP); although normal PCs have distinct gene expression profiles with MGUS and MM, at present it is still not possible to clearly distinguish MGUS from MM by GEP [28–32]. Studies have also looked at the association between gene expression patterns with molecular subtypes of MM [33–36]. It was shown that gene expression patterns are associated with primary IgH translocations and cyclin D gene expression. Various gene signatures associating with high risk have been proposed [33, 37–43], for instance the 70-gene signature (GEP70) developed by the University of Arkansas Medical Sciences. However, most of these signatures are not overlapping and have not been integrated into routine clinical care [6].

In addition, epigenetic deregulation was implicated in myelomagenesis. DNA methylation changes significantly during disease progression and could silence important tumor suppressor genes including SOCS1 [44–48]. Furthermore, the BM microenvironment also plays a crucial role in MM pathogenesis by promoting growth, survival, and drug resistance in MM cells. The adherence of MM cells to bone marrow stromal cells (BMSCs) increases the release of cytokines/growth factors, including IL-6 which activates the JAK-STAT signaling and promotes MM cell survival and proliferation [49–51].

However, despite enormous advances in understanding MM biology and emergence of novel therapeutics, the mechanism behind MM initiation and progression remains to be fully elucidated and the disease remains largely incurable [52]. Therefore, research effort on the elucidation of important role miRNA plays in MM may shed new light on MM pathobiology and identify novel biomarkers and therapeutic targets.

## 2. MicroRNAs

miRNAs are ~20-nucleotide genome-encoded RNAs highly conserved across different species and regulate most cellular processes [53]. As of 2013, more than 1800 miRNA precursors have been identified and deposited in the miRNA registry, miRBase. Each miRNA can target hundreds of different conserved or nonconserved genes. It has been estimated that a large proportion of the transcriptome (about 50% in humans) is subject to miRNA regulation [54, 55].

miRNAs are mostly transcribed by RNA polymerase II which generates long, capped, and polyadenylated precursors known as pri-miRNAs. Each pri-miRNA is subsequently processed by the microprocessor complex consisting of Drosha, a member of ribonuclease III enzyme family, and dsRNA-binding protein DGCR8/Pasha, resulting in a ~70-nucleotide precursor known as pre-miRNA which is actively exported by exportin 5 to the cytoplasm where it is cleaved near the terminal loop by another RNase III type endonuclease Dicer, generating a ~20-nucleotide miRNA duplex. Finally, the mature single-stranded miRNA product is loaded onto an Ago protein to form the effector complex called the RNA-induced silencing complex (RISC), and the other strand of the duplex is degraded. In RISC, the mature miRNA recognizes complementary sequence (usually in the 3′UTR region) of target to the seed sequence of miRNA (nucleotides 2–8 at 5′end), binds to the target mRNA, and regulates gene expression by translation repression or mRNA degradation depending on the degree of complementarity [54, 56, 57].

Deregulation of miRNAs has been associated with a plethora of human diseases including cancer. miRNAs regulate critical processes in tumor initiation and development by targeting oncogenes and/or tumor suppressor genes [58–60]. miRNA expression profiles have been shown to be able to classify human cancers with better accuracy than traditional GEP [61–64] and could serve as novel biomarkers for diagnosis, disease progression, and prognosis [61, 65–68]. Recently, miRNA deregulation has also been implicated in drug resistance in cancers including leukemia [69–71].

Deregulation of miRNA in cancer arises from both genomic and epigenetic changes [72]. Many human miRNA genes are located at cancer-associated fragile genomic locus that are subjected to frequent mutations [73–75]. Defect in miRNA biogenesis machinery is also shown to be affecting miRNA expression in cancer [76–78]. In addition, disruption of canonical miRNA/target binding sequence, for instance SNPs and altered splicing pattern of target mRNAs, may deregulate miRNA function and contribute to oncogenesis [79]. Recently, epigenetic aberrations, including DNA hypermethylation and/or histone modification, have emerged as a major cause in miRNA deregulation [80–82].

Increasing evidence suggests that miRNA deregulation is a hallmark of myeloma. This paper reviews the current literature on the roles miRNA play in MM pathobiology, prognosis, and therapy.

## 3. miRNA Deregulation in Different Stages of MM

As mentioned above, MM is characterized by multistep transformation and complex genomic aberrations both structurally and numerically. Many molecular subtypes of MM have been identified by GEP. Given that miRNA expression profile has the potential of improved accuracy over traditional GEP signatures and could represent novel biomarkers, a number of studies have looked at miRNA expression profiles in different stages and molecular subtypes of MM.

In a pioneering paper, Pichiorri et al. performed global miRNA expression profiles in samples from 5 MGUS patients, 10 MM and 4 normal PCs, and identified miRNA deregulated in MM and MGUS relative to normal PCs, including upregulation of miRNAs with known oncogenic activity such as miR-21, miR-106b~25 cluster, miR-181a, miR-181b, miR-32, and miR 17–92 cluster [75, 83, 84]. Among these, miR-32 and miR 17–92 are unique to overt MM but not MGUS.

Roccaro et al. conducted miRNA expression profiling in PCs from 15 relapsed/refractory MM samples, 3 MM cell lines, and 4 healthy donors. Unsupervised analysis showed clear separation of MM subjects and normal, although MM samples and cell lines were not separated. miRNAs deregulated in relapsed/refractory MM including downregulation of miR-15a and miR-16. Functional studies showed that these two tumor suppressor miRNAs inhibit proliferation and growth of MM cells* in vitro* and* in vivo*. At the same time, miR-15a and miR-16 decreased MM cell-induced proangiogenic activity on endothelial cells by reducing VEGF secretion from MM cells. Both miRNAs inhibited MM cells in the presence of BMSCs [85].

In a study focusing on PCL, Lionetti et al. compared miRNA expression profile in 18 primary PCL (pPCL) with 39 myeloma samples. Unsupervised analysis revealed a fairly distinct miRNA expression profile for pPCL relative to MM, as all PCL cases were clustered together and formed a main cluster with a few MM cases, whereas 4 normal samples were clustered as a distinct group [86].

Zhou et al. profiled miRNA expression in PCs from 52 newly diagnosed MM cases and 2 healthy donors and observed an elevated total miRNA expression level in MM. 39 miRNAs were upregulated in MM, including miR-18, miR-92a, miR-181a, miR-181b, miR-221, miR-222, and miR-99a which were consistent with previous reports. Only 1 miRNA, miR-370, was downregulated [87]. Chi et al. performed miRNA expression profiling in PCs from 33 MM patients, 5 MGUS cases, 4 HMCLs, and 5 healthy donors. Unsupervised analysis showed separation of normal samples from the rest. However, MM, MGUS, and HMCL were not separated into distinct clusters. Differentially expressed miRNAs between MM and normal were partially consistent with other reports [88].

These studies have explored the potential use of miRNA to distinguish MGUS from MM. Although some differentially expressed miRNAs between PCs from MGUS and MM were identified, no robust miRNA signature able to distinguish MGUS and MM were established. In a profiling study involving MM and pPCL, miRNA expression-based unsupervised clustering separated MM and pPCL samples with moderate success. Interestingly, majority of these differentially expression miRNAs showed same trend (upregulation or downregulation) from healthy controls, through MM, to pPCL. It is therefore tempting to hypothesize that the degree of miRNA deregulation correlates with the extent of tumor progression.

## 4. miRNA Deregulation in Different Molecular Subtypes of MM

Lionetti et al. profiled miRNA expression in MM subjects representative of 5 Translocation/Cyclin (TC) subtypes defined previously [34]. Unsupervised clustering loosely classified the samples according to their TC group. TC4 (MMSET-FGFR3) samples tightly clustered as a single branch, with upregulation of miR-99b, miR-125a-5p, and let-7e which belong to a cluster at 19q13.33. All samples in the TC5 group (MAF or MAFB translocation) except one were also tightly clustered. Interestingly, miRNAs specifically upregulated in TC5 group include miR-99a, let-7c, and miR-125b-2 which belong to a paralogous miRNA cluster of the three in TC4 [89]. The upregulation of all or some members of the miR-99b, miR-125a-5p, and let-7e cluster in t(4; 14) MM was also observed in three other studies including the study on pPCL [86, 88, 90].

Gutiérrez et al. compared 60 MM patients with 5 healthy donors and identified downregulation of 11 miRNAs. Unsupervised analysis did not classify samples into clearly separated clusters according to molecular subtype, although the four samples with MAF translocations were tightly clustered [91]. In another analysis with overlapping samples, unsupervised analyses based on miRNA expression in MM identified unique clusters not associated with chromosomal abnormalities; one cluster is comprised of upregulated miRNAs including miR-21, members of the miR-17~92, and miR-106b~25 clusters, although the biological relevance of the clustering pattern remained elusive.

Studies have also looked at the association of miRNA deregulation and other genetic features of MM. Pichiorri et al. compared miRNA expression profile between HMCLs with wild-type (WT) TP53 and those with mutant TP53. Higher expression of miR-192, miR-194, and miR-215 in HMCLs were observed in HMCLs with WT TP53, as well as miR-34a which is a well-documented TP53 target. miR-192, miR-194, and miR-215 could also be induced by nutlin-3a treatment in MM cell harboring WT TP53 but not mutant TP53, suggesting that these miRNAs were regulated by p53. The authors went on to show that these miRNAs were transcriptionally activated by p53 and target MDM2, forming a positive feedback loop. These miRNAs exhibited anti-MM functions in a p53 dependent manner and could sensitize TP53 WT cells to MDM2 inhibitors [92].

Rio-Machin et al. examined miRNA expression profiles in hyperdiploid and nonhyperdiploid MM. Downregulation of specific miRNAs including miR-425, miR-152, and miR-24 was observed in hyperdiploid MM. Intriguingly, downregulation of these miRNAs was accompanied by a concomitant upregulation of their targets CCND1, TACC3, MAFB, FGFR3, and MYC, which were also the oncogenes upregulated by the most recurrent IgH translocations in nonhyperdiploid MM. This suggested that miRNA deregulation could be the mechanism behind cyclin D as a unifying feature in both nonhyperdiploid and hyperdiploid MM [93].

These studies have demonstrated that miRNA expression tends to correlate with molecular subtypes of MM, most notably with t(4; 14) and t(14; 16) translocations. Interestingly, miR-99b, miR-125a, and let-7e which belong to a cluster at 19q13.33 were consistently associated with t(4; 14) in multiple studies. However the cause and effect relationships for these are still not clear.

## 5. miRNA Deregulation and Clinical Parameters in MM

A number of studies attempted to correlate miRNA with clinical parameters such as risk group and survival. Roccaro et al. identified a significant reduction of miR-15a level in MM patients in ISS II and III groups as compared to ISS I group, which was confirmed by another study [94], consistent with its function as a tumor suppressor. Meanwhile, miR-181a and miR-181b were expressed at higher levels in ISS II and III groups [85]. Zhou et al. showed that globally elevated miRNA expression was associated with higher GEP70 risk score and proliferation index, suggesting that high expression level of miRNA might confer an inferior clinical outcome. In addition, unsupervised clustering of miRNA expression profiles stratified patients according to risk, although no association was found with proliferation index [87]. Chi et al. identified differentially expressed miRNAs between light chain only MM and nonlight chain only MM, IgG and IgA-type MM, as well as patients with event-free survival (EFS, median follow-up = 20 months) and those who relapsed/died in this interval. These differentially expressed miRNAs were shown to have good prediction accuracy [88].

Wu et al. identified that higher expression of three miRNAs miR-886-5p, miR-17, and miR-18a was significantly associated with shorter overall survival of patients. It was noteworthy that miR-17 and miR-18a were members of the oncogenic miR-17~92 cluster. Furthermore, miR-886-5p and miR-17 formed a robust outcome classifier which could improve the ISS/FISH based risk stratification independent of previously validated GEP signatures [90].

In the pPCL study, Lionetti et al. identified 4 miRNAs (miR-106b, miR-497, miR-181b, and miR-181a*) upregulated in pPCL patients not responding to initial therapy consisting of lenalidomide and low-dose dexamethasone, compared to responders. Consistent with their oncogenic roles, miR-106b and miR-181b were already reported to be upregulated in MM cells compared to normal PCs [84]. Moreover, the expressions of miR-22 and miR-146a were identified to be associated with progression-free survival (PFS) while the expressions of miR-92a and miR-330-3p were identified to be associated with overall survival (OS) of pPCL patients, demonstrating their relevance in clinical prognostication in this aggressive form of plasma cell dyscrasia [86].

Besides miRNA expression, miRSNPs (SNPs in miRNA genes, miRNA processing machinery, or miRNA target genes) could affect the final level and function of miRNAs and could be clinically important. Two miRSNPs that had prognostic impact after autologous stem cell transplant (ASCT) were identified, one in the 3′UTR of a miRNA target gene, KRT81, another in XPO5, a crucial gene in the miRNA biogenesis pathway. Patients with different SNPs in either of these two miRSNPs showed significant difference in OS [95].

A number of recent studies have looked into circulating miRNAs for their potential as novel biomarkers. In one study comparing plasma miRNA profile between MM patients and healthy controls, six miRNAs (miR-148a, miR-181a, miR-20a, miR-221, miR-625, and miR-99b) were found upregulated in MM patients. Notably, miR-181a, miR-20a, miR-221, and miR-625 were identified previously to be unregulated in the plasma cells of MM or MGUS [84, 87, 88]. miR-99b was upregulated in t(4; 14) MM, consistent with previous reports [88–90]. Moreover, higher plasma levels of miR-20a and miR-148a were found to correlate with a shorter relapse-free survival [96].

Other deregulated serum/plasma circulating miRNAs have been identified, including miR-92a whose expression was lower in MM [97] and miR-29a which was expressed at a higher level in serum of MM patients [98]. miR-1308 and miR-720 could distinguish MGUS and MM patients from healthy controls [99]. Lower levels of miR-744 and let-7e were associated with shorter OS and remission [100].

These studies have linked miRNAs with clinical parameters, although the results are different from each other and no consistent miRNA-based biomarker is reported. Moreover, few studies have compared the usefulness of miRNA-based biomarkers with the current standard of care, except one study which identified a miRNA-based OS classifier that performed better than traditional ISS/FISH based method and outperformed existing GEP-based models in multivariate analysis [90]. Further validation of this prognostic signature in other cohort of patients is needed to ascertain its clinical utility. Similarly, while these studies demonstrated the feasibility of detecting miRNA in the serum and their potential clinical relevance, these findings needs to be further validated. Therefore, the exact clinical utility of measuring miRNA in serum is still unclear.

## 6. Interaction of miRNA and Current Therapeutic Agents in MM

In one of the earlier studies, Munker et al. studied miRNA expression profiles between MM cell lines with acquired resistance to doxorubicin or melphalan and the respective parental cells. Differentially expressed miRNAs include miR-21 and miR-181a/b, although their functional link to the resistance was not clear [101]. Wang et al. showed that adherence of MM cells to BMSCs upregulates miR-21 which resulted in decreased cytotoxicity to dexamethasone, doxorubicin, and bortezomib. Inhibition of miR-21 sensitized cells to dexamethasone and doxorubicin but not Bortezomib [102]. Tessel et al. identified a link between miR-130b and glucocorticoid resistance in MM, where miR-130b inhibited dexamethasone-induced apoptosis [103]. Similar result was observed in another study in which miR-125b was shown to attenuate dexamethasone-induced cell death in MM [104].

Hao et al. showed that the reduced sensitivity of MM cells to bortezomib and melphalan after coculture with BMSCs is at least partially due to inhibition of tumor suppressor miR-15a [105], while another miRNA, miR-29b, could sensitize MM cells to bortezomib-induced apoptosis and exerts anti-MM activity both in cultured MM cells and in MM xenografts in mice. miR-29b showed wide variation of expression in MM and the expression was decreased with the presence of BMSCs, again demonstrating the critical role of BMSCs in promoting drug resistance and survival of MM cells. Notably, miR-29b mimic was able to overcome the protective role of BMSCs in an* in vivo* model [106].

Tian et al. identified miR-33b as an important mediator for the anti-MM function of MLN2238, a novel, orally active proteasome inhibitor. Inhibited in MM cells, miR-33b was upregulated by MLN2238, but not by other agents including dexamethasone, lenalidomide, and SAHA. Upregulation of miR-33b decreased MM cell viability, migration, and colony formation and increased apoptosis and sensitivity of MM cells to MLN2238 treatment. Notably, MLN2238 induced miR-33b even in the presence of BMSCs, and introduction of miR-33b partially blocked the protective effect of BMSCs on MM cells [107].

The evidence therefore suggests that a number of miRNA may be involved in therapeutic resistance mediated by stromal interaction. These may offer potential strategies to overcome drug resistance in myeloma. However, it is still not clear what the pathways affected by these miRNAs that may be critical in mediating drug resistance are.

## 7. The Interplay between miRNA and Epigenetics in MM

Aberrant miRNA expression or function in cancer can be attributed to various mechanisms involving both genomic and epigenetic aberrations. It has been observed in MM that miRNA expression could be disrupted by deregulation of miRNA host genes, copy number (CN) at miRNA-containing genomic locus [86, 89, 108, 109], abnormalities in miRNA biogenesis pathways [87], and abnormal activity of transcription factors [110]. However, it seems that the most important mechanism behind aberrant miRNA deregulation is epigenetic alterations, including abnormal DNA methylation and histone modifications [111–114]. Inactivation by methylation of all three members of miR-34 family tumor suppressor miRNAs was identified in MM [115–119]. Di Martino et al. provided a proof-of-principle that formulated that lipid emulsion delivery of synthetic miR-34a has therapeutic activity in preclinical, TP53 mutant xenograft models in MM [120]. Recently, the same group used a nanotechnology-based delivery system for miR-34a delivery and demonstrated similar anti-MM effect in tumor xenograft [119]. The promoter of the other two members of the miR-34 family, miR-34b and miR-34c, was not methylated in normal PCs, methylated in about 5.3% at MM diagnosed and increased frequency to more than half of relapsed/progressed MM patients. Functionally, restoration of miR-34b exhibited anti-MM activity* in vitro* [117]. Similar to miR-34a, downregulation of p53-inducible miR-192, miR-194, and miR-215 was attributed to promoter hypermethylation, which would impair the p53/MDM2 loop and favors MM development [92].

Another tumor suppressor, miR-203, was identified to be methylated at its promoter region in MM but not in normal PCs, and transfection of its precursor inhibited proliferation of MM cells [116]. Moreover, increasing frequency of promoter methylation in MM than MGUS for miR-129-2 was observed [118]. Aberrant DNA methylation could also explain the downregulation of other miRNAs in MM, including miR-214 which inhibited cell proliferation when overexpressed in MM cells. Consistent with the epigenetic silencing hypothesis, the level of miR-214 could be increased by treatment with DNA demethylating agent 5′aza-2′-deoxycytidine [91, 121].

Besides aberrant DNA methylation, miRNA deregulation by histone modification had also been documented in MM. Min et al. showed that in t(4; 14) myeloma, repression of miR-126* expression, contributed to c-Myc upregulation and enhanced proliferation of MM cells. The downregulation of miR-126* was due to heterochromatin modification by MMSET [122].

It has been shown that miRNAs themselves can regulate the epigenetic machinery by directly targeting their enzymatic mediators such as DNMTs [123]. One such miRNA in MM is miR-29b, whose tumor suppressor property was earlier discussed. miR-29b targets* de novo* methyltransferases DNMT3A and DNMT3B mRNAs and reduces global DNA methylation in MM cells and therefore could restore expression of tumor suppressor genes silenced by hypermethylation such as SOCS1 [124].

Various mechanisms behind miRNA deregulation have been identified. Epigenetic aberrations, in particular abnormal DNA methylation at miRNA promoter regions, seem to be widespread and critical in silencing tumor suppressor miRNAs such as miRs-192, -194, -215, and miR-34 family. Our group has conducted genome-wide analysis of miRNAs silenced by DNA methylation and functionally studies miRNAs upregulated by demethylating treatment. Apart from known miRNAs that are epigenetically silenced, our study has revealed novel tumor suppressor miRNAs relevant in MM pathobiology (unpublished data). Again it highlights the importance of the epigenetic-miRNA regulatory network in MM.

## 8. miRNA and IL6-STAT3 Signaling in MM

The IL6-JAK-STAT axis is a major mediator of growth/survival promoting effect on MM conferred by BM microenvironment. Secreted by the BMSCs, IL6 binds to its receptor and activates JAK kinase, which in turn activates STAT3. The activated STAT3 translocates into the nucleus and activates transcription of genes that promote growth, proliferation, and survival of MM cells. The IL6-STAT3 signaling pathway is tightly controlled by SOCS proteins which binds to JAK and inhibits receptor phosphorylation and STAT3 activation [49–51]. However, SOCS1 is often silenced by promoter hypermethylation in MM, leading to enhanced IL6-STAT signaling [44, 45].

**Figure 1:**
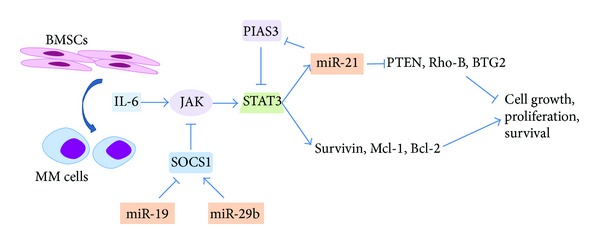
miRNA and IL6-STAT3 signaling in MM. miR-21 is upregulated upon adherence of MM cells to BMSCs. It can be directly induced by STAT3 and contribute to the oncogenic potential of STAT3. At the same time, miR-21 can indirectly induce STAT3 by targeting PIAS3, a STAT3 inhibitor, forming a positive feedback loop. miR-19 has been shown to promote STAT3 signalling by repressing SOCS1. miR-29b could demethylate SOCS1 by targeting DNMTs, leading to upregulation of SOCS1, and negatively regulates IL6-STAT3 signalling.

Studies have revealed roles of miRNAs as important regulators and mediators of this axis in MM. miR-21 is upregulated upon adherence of MM cells to BMSCs [102, 125]. It can be directly induced by STAT3 and contribute to the oncogenic potential of STAT3 [83]. At the same time, miR-21 can indirectly induce STAT3 by targeting PIAS3, a STAT3 inhibitor, forming a positive feedback loop [126]. miR-19 has been shown to promote STAT3 signaling by repressing SOCS1 [84]. Interestingly, miR-29b could demethylate SOCS1 by targeting DNMTs, leading to upregulation of SOCS1 levels and negatively regulates IL6-STAT3 signaling [45, 127] (Figure 1).

## 9. miRNA and p53 in MM

**Figure 2:**
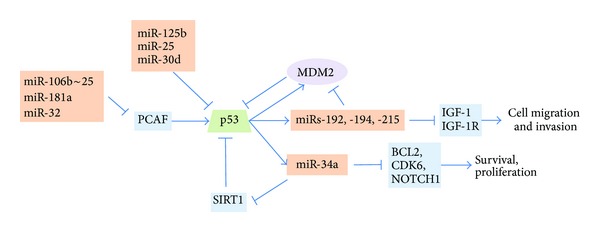
miRNA and p53 in MM. P53 can be directly targeted by miR-125b, miR-25, and miR-30d and indirectly targeted by miR-106b~25 cluster, miR-32, miR-181a which target PCAF, a positive regulator of p53. Upregulation of these miRNAs in MM were observed in multiple studies. On the other hand, p53 transcriptionally induces miRs-192, -194, and -215 which target MDM2 and miR-34a which target SIRT1. Both pathways lead to upregulation of p53, forming two positive feedback loops. Deregulation of these miRNAs leads to compromised p53 tumor suppressor pathway and favors oncogenesis.

P53 mutation in newly diagnosed MM is rare and its frequency increases with disease progression. One copy loss of p53 by FISH has been uniformly recognized to be an adverse prognostic factor of MM [6]. It has been shown that p53 can be directly targeted by miR-125b, miR-25 and miR-30d [104, 128], and indirectly targeted by miR-106b~25 cluster, miR-32, and miR-181a which target PCAF, a positive regulator of p53 [84]. Upregulation of these miRNAs in MM was observed in multiple studies. On the other hand, p53 transcriptionally induces miRs-192, -194, and -215 which target MDM2 [92], and miR-34a which target SIRT1 [104]. Both pathways lead to upregulation of p53, forming two positive feedback loops. Deregulation of these miRNAs leads to compromised p53 tumor suppressor pathway and favors oncogenesis (Figure 2).

## 10. Key miRNAs with Therapeutic Potential in MM

MiRNA possesses promising therapeutic potential in cancer because it can target many important genes or pathways at the same time. A number of deregulated miRNAs are consistently identified and their important functions are demonstrated (see Table 1). Discussed below are some of the miRNAs that have demonstrated the most promising therapeutic potential.

### 10.1. MiR-29b and miR-21

MiR-29b has been shown to inhibit tumor growth in HMCLs and in mouse. It can also contribute to the antitumor activity of Bortezomib and potentiates Bortezomib-induced apoptosis when used together [106]. The tumor suppressor property of miR-29b may be partially explained by its inhibition on IL6-JAK-STAT3 signaling via targeting DNMTs and subsequent demethylation and activation of SOCS1 [124]. Moreover, it target proangiogenic factors including VEGFA, inhibits migration, and negatively regulates osteoclast activity which may alleviate lytic bone disease [127, 129, 130].

MiR-21 has been identified as a direct target of STAT3 that potentiates IL6-STAT3 signaling [83]. Upregulation of miR-21 upon adherence to BM has been shown for HMCLs and primary samples, which may be explained by enhanced IL6-STAT signaling. Targeting miR-21 inhibits* in vitro* and* in vivo* MM growth even in the context of BM and could synergize with chemotherapeutic agents dexamethasone and doxorubicin [102, 125].

### 10.2. miRs-192, -194, -215, and miR-34a

17p deletions, mostly including TP53, have been unequivocally identified as a predictor for worse prognosis in MM. miRNAs have been implicated in p53 pathway. In particular, miRs-192, -194, -215, and miR-34a have been extensively studied. All these four miRNAs are direct transcriptional targets of p53, reversely; these miRNAs indirectly induce p53, forming two positive feedback loops and participating in the regulatory balance of p53. Promoter hypermethylation of these miRNAs has been found in MM and could lead to their silencing. Reexpressing miRs-192, 194, and 215 leads to downregulation of their direct target MDM2 and could sensitize TP53 WT cells to pharmacological inhibition of MDM2* in vitro* and* in vivo*. In addition, miRs 192, -194, and -215 could inhibit migration and invasion of MM cells by targeting IGF1 and IGF1R [92]. miR-34a is an established tumor suppressor in cancer. In MM, miR-34 has demonstrated excellent antitumor activity in preclinical models. Transient and prolonged expression of miR-34a inhibited tumor growth both* in vitro* and* in vivo*. In mouse models, both intramural injection and systemic delivery of miR-34a in lipid particles inhibited tumor growth. Importantly, miR-34a could overcome the BM-dependent protective effect on MM cells, as demonstrated by a novel 3D system [119, 120]. It should be noted that most of the cells used in miR-34a study are TP53 mutant, suggesting that patients with p53 inactivation may in particular benefit from miR-34a replacement therapy.

### 10.3. miR-15a

MiR-15a has been closely associated with bone marrow microenvironment. The secretion of tumor suppressive miR-15a in exosomes by normal BM is reduced in tumor microenvironment, producing a permissible environment for tumorigenesis and reduces sensitivity to bortezomib and melphalan [105, 131]. Restoring miR-15 inhibited AKT, NF*κ*B activity, and VEGF and exerted antitumor effects even in the context of BM [85, 94].

## 11. Conclusion

In conclusion, miRNAs have emerged as important players in the pathobiology of MM and have potential in improving clinical practice. Future research should focus on the validation of miRNA signatures and the integration of validated signatures in clinical practice for better disease classification, prognostication, and prescription. At the same time, miRNAs with the most promising therapeutic potential should be moved into the pipeline of clinical development, as single agents or in combination with current therapy, guided by improved understanding of the disease.

## Figures and Tables

**Table 1:** Selected miRNA deregulations in MM.

miRNA	Deregulation in MM (versus normal control unless specified)	Targets/function/clinical relevance	Association with clinical parameters
21	Upregulated in MM and MGUS [84, 88]; upregulated in primary PCL versus MM [86]	PIAS3 [126], PTEN [125], Rho-B [102, 125], BTG2 [125]; induced by STAT3 in response to IL-6 [83]	

221, 222	221: upregulated in MM [84, 87, 88]; 222: upregulated in MGUS [84, 88], MM [87, 88]; 221 and 222: upregulated in relapse/refractory MM [85]	p27Kip1, PUMA, PTEN and p57Kip2 [132]	

17-92 cluster (17, 18a, 19a, 19b-1, 20a, 92a)	Upregulated in MM but not in MGUS [84]; upregulated in MM [87, 88, 133]; Positively regulated by Myc [110]; lower plasma miR-92a level in MM than MGUS, SMM and normal.[97]	BIM, SOCS1 [84]; P21 [87]	Higher 92a was associated with shorter OS [86]; Higher 17, 20a, and 92-1 were associated with shorter PFS [110, 134]; higher 17 and 18a were associated with shorter OS [90]; Higher plasma miR-20a was associated with shorter relapse-free survival [96]

106b~25 cluster (106b, 93, 25)	Upregulated in MM and MGUS [84]; upregulated in MM [133]; miR-25 is overexpressed in MM [128]	PCAF [84]	miR-106b was correlated with treatment response [86]

181a/b	Upregulated in MM and MGUS [84]; upregulated in MM [85, 87, 88]	PCAF [84]	miR-181a* and miR-181b were correlated with treatment response [86]

25, 30d, 125b	Upregulated in MM [84, 87, 128]	P53 [128]	

32	Upregulated in MM not in MGUS [84]	PCAF [84]	

15a and 16-1	Decreased in relapsed/refractory MM [85]; decreased in MM [88]; decreased in patients with 13del as compared to those without [88]; expressed in MM independent of chr13 status [134, 135]	AKT3, rpS6, MAP-kinases, MAP3KIP, VEGF [85]	Decreased in patients with ISS stage III [94]; higher expression correlates with shorter PFS [134]

192, 194, 215	Downregulated in MM by promoter hypermethylation [92]	Activated by TP53 and targeted MDM2, IGF1, IGF1R [92]	

34 family	Downregulated in MM by promoter hypermethylation [115, 117]	BCL2, CDK6 and NOTCH1 [120]	

203	Downregulated in MM [91, 135]; decreased in MGUS and MM by promoter hypermethylation [116]	CREB1 [116]	

33b	Downregulated in MM [107]	Involved in MLN2238-induced apoptotic signaling in MM cells [107]	

29b	Wide variation of expression in MM and further decreased with the presence of BMSCs [106]	DNMT3A/B [124], CDK6 [106], MCL-1 [106, 129], Sp1 [106]; Targeted VEGFA, IL8; induced SOCS1 [127]	

425, 152, 24	Downregulated in hyperdiploid MM versus nonhyperdiploid MM [93]	CCND1, TACC3, MAFB, FGFR3, MYC [93]	

214	Downregulated in MM versus normal PCs [91], possibly by methylation [121]	PSMD10 [121]	

126*	Downregulated in t(4;14) MM cells; inhibited by MMSET by heterochromatin modification [122]	c-Myc [122]	
